# A *Bacillus thuringiensis* Chitin-Binding Protein is Involved in Insect Peritrophic Matrix Adhesion and Takes Part in the Infection Process

**DOI:** 10.3390/toxins12040252

**Published:** 2020-04-13

**Authors:** Jiaxin Qin, Zongxing Tong, Yiling Zhan, Christophe Buisson, Fuping Song, Kanglai He, Christina Nielsen-LeRoux, Shuyuan Guo

**Affiliations:** 1 School of Life Science, Beijing Institute of Technology, Beijing 100081, China; 2 State Key Laboratory for Biology of Plant Diseases and Insect Pests, Institute of Plant Protection, Chinese Academy of Agricultural Sciences, Beijing 100193, China; 3 Micalis Institute, INRAE, AgroParisTech, Université Paris-Saclay, 78350 Jouy-en-Josas, France

**Keywords:** *Bacillus thuringiensis*, chitin-binding protein, adhesion, peritrophic matrix

## Abstract

*Bacillus thuringiensis* (Bt) is used for insect pest control, and its larvicidal activity is primarily attributed to Cry toxins. Other factors participate in infection, and limited information is available regarding factors acting on the peritrophic matrix (PM). This study aimed to investigate the role of a Bt chitin-binding protein (CBPA) that had been previously shown to be expressed at pH 9 *in vitro* and could therefore be expressed in the alkaline gut of lepidopteron larvae. A ∆cbpA mutant was generated that was 10-fold less virulent than wild-type Bt HD73 towards *Ostrinia furnacalis* neonate larvae, indicating its important role in infection. Purified recombinant *Escherichia coli* CBPA was shown to have a chitin affinity, thus indicating a possible interaction with the chitin-rich PM. A translational GFP–CBPA fusion elucidated the localization of CBPA on the bacterial surface, and the transcriptional activity of the promoter P*cbpA* was immediately induced and confirmed at pH 9. Next, in order to connect surface expression and possible *in vivo* gut activity*,* last instar *Galleria* *mellonella* (Gm) larvae (not susceptible to Bt HD-73) were used as a model to follow CBPA in gut expression, bacterial transit, and PM adhesion. CBPA-GFP was quickly expressed in the Gm gut lumen, and more Bt HD73 strain bacteria adhered to the PM than those of the ∆cbpA mutant strain. Therefore, CBPA may help to retain the bacteria, via the PM binding, close to the gut surface and thus takes part in the early steps of Bt gut interactions.

## 1. Introduction

*Bacillus thuringiensis* (Bt) is a prominent insect pathogen of the *Bacillus cereus* group. Several strains are used worldwide as microbial control agents against major agricultural and forest insect pests [1]. The primary insecticidal factors of Bt are the Cry toxins, which comprise parasporal protein crystals produced by Bt, and numerous studies have focused on the structural resolution of the crystals and on the mode of action of Cry toxins [2]. As an insect pathogen [3], the roles of Bt itself in pathogenesis has been much less investigated and may depend on the insect species, larval stage, and Bt strain.

For pathogenic bacteria, the successful establishment of infection generally requires adhesion, colonization, and host cell degradation or active invasion. The capacity for host cell and tissue adherence is a key feature of pathogenic bacteria [4,5]. Orally acting entomopathogenic bacteria including Bt face the peritrophic matrix (PM) just after ingestion. The PM is an important component of the insect digestive tract: It serves both as a physical barrier to separate food particles, digestive enzymes, and pathogens, and it serves as a biochemical barrier, sequestering or even inactivating ingested toxins [6]. Therefore, Bt must bypass the PM barrier to establish persistent infections [7] in order to develop and complete the process of infection and life cycle, ending with sporulation in the insect cadaver [8,9].

The PM is a chitin- and glycoprotein-rich matrix, separating intestinal cells from the gut content [10]. Chitin is a linear polymer of N-acetylglucosamine (GlcNAc) linked via β-1,4 linkage [11]. Chitinases can hydrolyze chitin, thus fragmenting the PM and suggesting that chitinases may be part of the enzymes involved in the degradation of the PM [12]. Chitinases can enhance the insecticidal activity of Bt, irrespective of chitinase activity derived from a chromosomal gene, the co-expression of chitinase with a Cry toxin gene, or even from the addition of commercial chitinases [13,14,15,16]. The most probable role of the endogenous chitinases of Bt is to weaken the integrity of the insect PM, facilitating the better access of the bacterial toxins and the bacteria to the gut epithelia [17].

Chitin-binding proteins (CBP) are present in numerous microorganisms. They belong to the 14, 18, or 33 families of the carbohydrate-binding domain proteins [18]. Various microorganisms simultaneously synthesize chitinases and CBPs [19]. The subcellular localization of CBP differs in accordance with the organism, most of them being secreted proteins [19,20]. Structural analyses have revealed the presence of the aromatic amino acid residues exposed on most CBPs, which play an important role in substrate binding [21,22,23,24,25]. From viruses to invertebrate organisms, CBP participates in various biological processes in different species, such as antifungal activity [26], synergistic effects with chitinase [19], and the detection of hydrophobic surfaces [27].

The alkaline pH of the midgut—in lepidopteron larvae, in particular—is needed for Bt to exert insecticidal activity, since an alkaline pH permits the solubilization of several Cry toxin crystals [1]. Hence, it is important for bacteria to adhere to host tissue and survive in this alkaline environment in order to pursue infection. Transcriptome gene microarray data previously indicated that *cbp3189* is up-regulated more than eight-fold after alkaline induction [28]. The protein encoded by *cbp3189* is referred to as chitin binding protein A (CBPA) in this study. CBPA is a conserved protein in Bt strains, as its amino acid sequence homology in 34 different Bt strains is greater than 97%, and, among them, nine strains have a 100% sequence identity. This gene encodes a protein containing a signal peptide and a transmembrane structure, and localization prediction has revealed that CBPA may be localized on the bacterial cell wall [29].

In this study, we addressed several questions related to the function, expression, and localization of CBPA. First, to determine whether CBPA plays a role during infection, a ∆cbpA mutant was constructed and assessed for virulence in *Ostrinia furnacalis* and *Galleria mellonella* larvae. Further, its subcellular localization in Bt and the activity of its promoter were assessed during in vitro growth. CBPA was expressed and purified from *Escherichia coli* through binding to chitin beads, and it was further analyzed for chitinase activity. Finally, the interaction of CBPA with the gut and the PM in vivo was assessed in *G*. *mellonella*, with a focus on the early stages of infection. Our results may provide functional insights into the role of CBPA in adhesion to the PM, thus improving the current understanding of the mode of action of Bt insecticidal strains, particularly for insects where the action of Cry toxins, spores, and out-grown bacteria are important for full virulence.

## 2. Results

### 2.1. ∆cbpA Mutant, ∆cbpA::cbpA-Complemented Strain Construction

**Figure 1:**
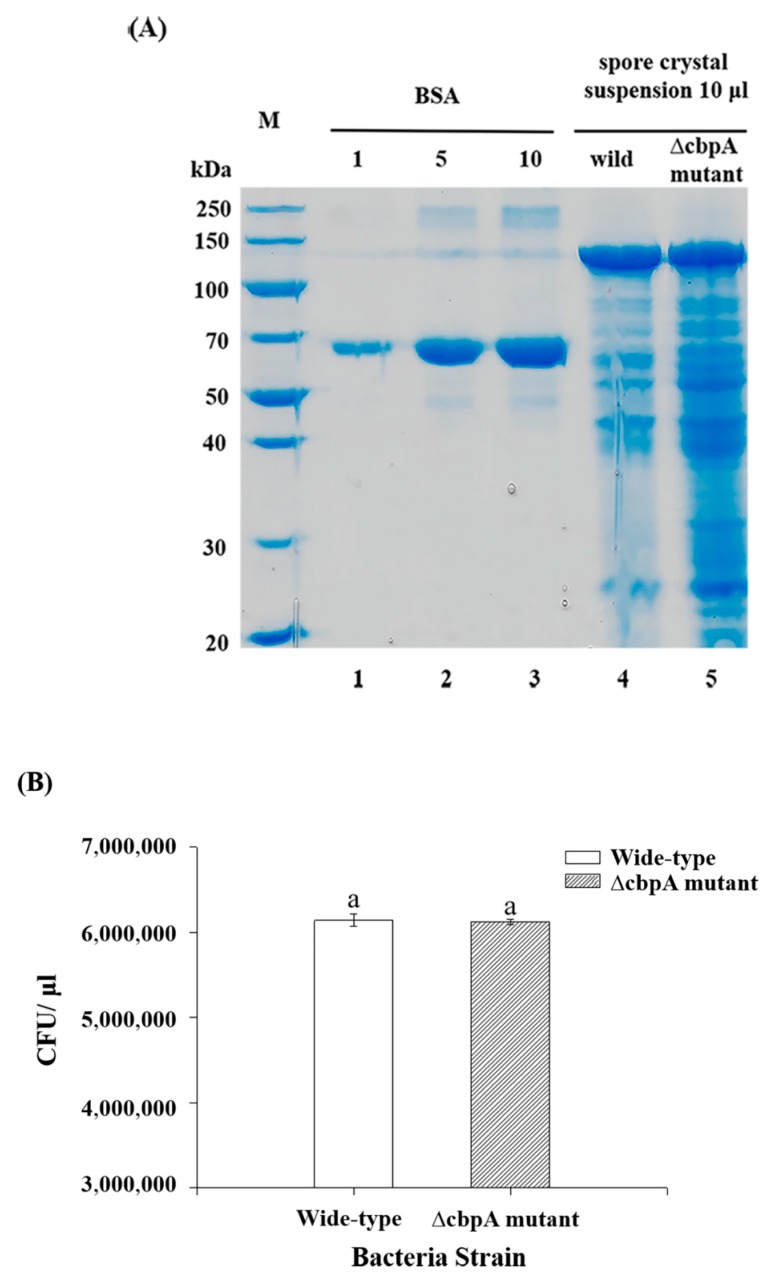
Comparison of Cry protein expression and spore formation in *Bacillus thuringiensis* (Bt) HD73 wild-type strain and ∆cbpA strain. (**A**) Cry protein production analyzed via SDS-PAGE. (**B**) Spore counts. 1. Bovine serum albumin (BSA) (1 µg); 2. BSA (5 µg); 3. BSA (10 µg); 4. Cry1Ac protein in wide-type (10-µL spore crystal suspension); 5. Cry1Ac protein in deletion mutant (10-µL spore crystal suspension). “a” indicates there was no significant difference (*p* > 0.05).

To determine the role of CBPA in insect infection, an interruption mutant strain ∆cbpA mutant was constructed via homologous recombination. Cry1Ac protein expression levels were not changed in the ∆cbpA mutant in comparison with wild-type HD73 upon protein quantification (Figure 1A). Spore count results showed that the wild-type strain and ∆cbpA mutant contained equal CFU values (Figure 1), indicating that the absence of *cbpA* did not influence spore formation.

### 2.2. Role of CBPA in Ostrinia Furnacalis and Galleria Mellonella Mortality

**Figure 2:**
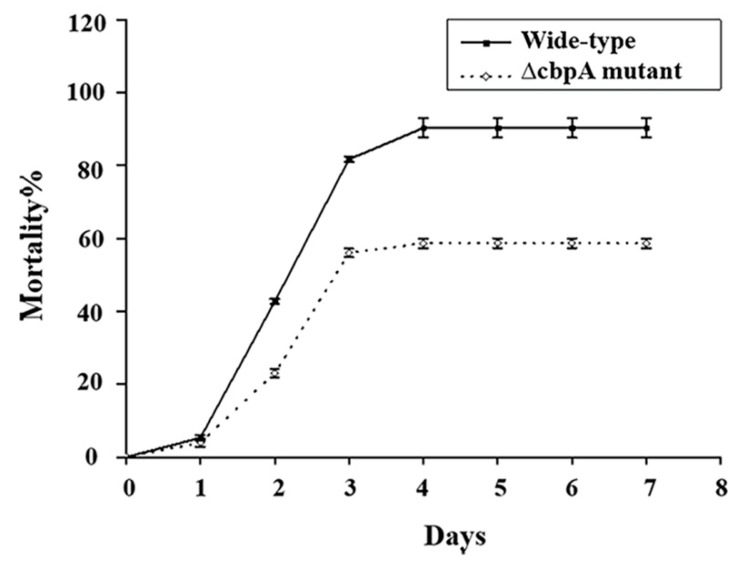
Comparison of mortality rates between the wild-type HD73 strain and the ΔcbpA mutant strain against the Asian corn borer (Cry protein: 2.5µg/g diet; spore: 2.7 × 10^9^/g diet).

As the Bt HD73 produces Cry1Ac that is toxic against the Asian corn borer (*Ostrina*), we selected this insect to evaluate the difference in mortality of larvae fed a diet supplemented with spore-crystal suspensions of wild-type or ∆cbpA mutant HD73 at various concentrations. Mortality induced by the ∆cbpA mutant was lower than that of the wild-type strain (Figure 2 and Table 1). Figure 2 shows the comparison of mortality rates between the wild-type HD73 strain and the ∆cbpA mutant strain for seven days post-feeding. For both strains, almost no change in mortality was observed after the fourth day of feeding. Mortality rates on the seventh day after feeding with seven different concentrations are listed in Table 1. The mortality between the wild-type and mutant strains was significantly different from the third concentration (Cry1Ac protein: 0.05 µg/g; spore: 5.4 × 10^7^/g).

Further, the difference in LC_50_ was evaluated by administering larvae with a diet supplemented with spores of the HD73 wild-type strain, the ∆cbpA mutant, or ∆*cbpA*::*cbpA*-complemented strains at different doses (10^4^–10^8^ CFU/per gram diet) and with the concentration of Cry1Ac at 0.01 µg/g diet. Table 2 shows the LC_50_ values of the three strains. Deionized water was used as the negative control, and the mortality was 2% in this control. The inferred larval mortality of the ∆cbpA mutant was significantly lower than that of the wild-type strain, while that of the complemented strain reverted to levels of the wild-type strain. These data indicate that CBPA significantly contributes to Bt virulence in *Ostrinia*.

To further investigate the role of CBPA, we tested the mutant for virulence towards *G. mellonella*; this insect needs bacteria associated with Cry1Ca for complete virulence in the synergy model [30,31]. Therefore, *G. mellonella* is a suitable model to elucidate the role of Bt and *B*. *cereus* chromosomal carried factors, and large last instars are easy to manipulate for accurate feeding and for dissection. Infections were induced through controlled force-feeding at a dose of 5 × 10^6^ mid log-phase vegetative bacteria (OD_600_ = 1) or with spores associated with 3 µg of activated Cry1Ca toxin for each larva, as described previously [32]. The results (Figure S1) showed no differences in mortality between infection with wild-type HD73 and the ∆cbpA mutant strains under all tested conditions. Indeed, no mortality was observed with spores or log-phase bacteria alone, and 90–100% mortality was observed when associated with 3 µg of Cry1Ca for both strains. Therefore, under these infection conditions, no clear role of CBPA was elucidated in Bt HD-73 mortality towards *G. mellonella* last instars.

### 2.3. Localization of CBPA in Bt HD-73

**Figure 3:**
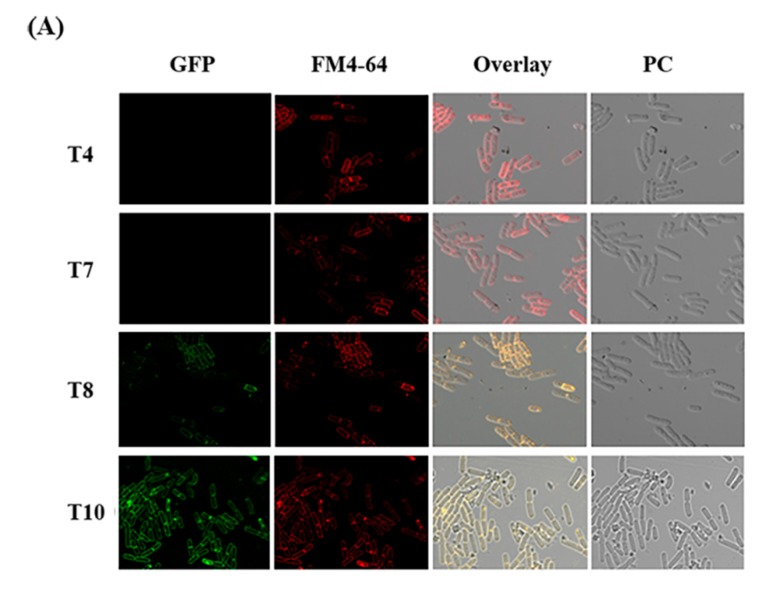

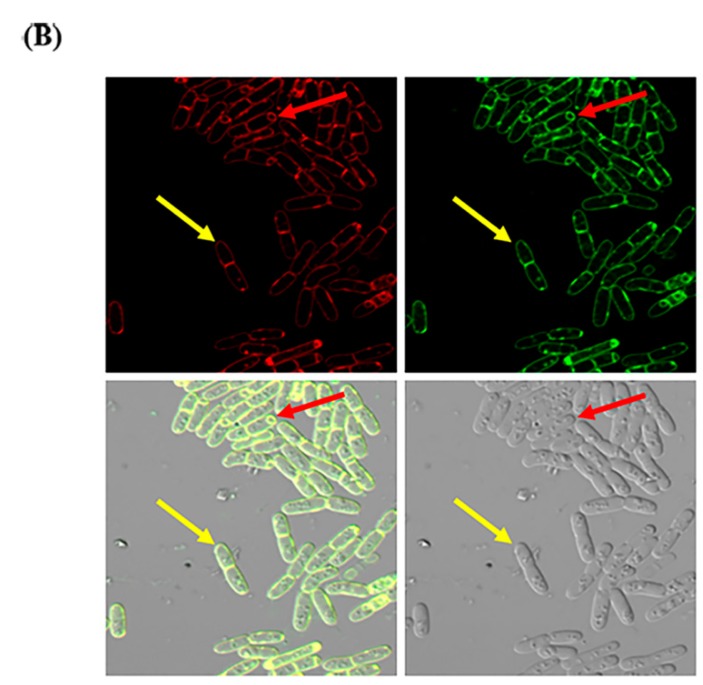
Green fluorescence detection of the chitin binding protein A (CBPA)–GFP fusion at different stages of culturing, observed via laser-scanning confocal microscopy. (**A**) Stages T4, T7, T8, and T10. (**B**) Localization of CBPA (T12). Yellow arrow denotes green fluorescence on the bacterial cell surface. Red arrow denotes green fluorescence on the spore surface. GFP (green fluorescent protein) signal in the bacterial cytosol. FM 4-64, (red fluorescent signal of bacterial membrane stain). The overlay shows green and red fluorescent signals. PC: phase-contrast microscopy.

GFP-conjugated CBPA was used to investigate the subcellular localization of CBPA in HD73 bacteria cells. Samples were harvested at different growth stages (T4, T7, T8, and T10 after the onset of the stationary phase). GFP expression was visualized via laser-scanning confocal microscopy. The cell membrane was stained with FM 4-64 dye solution. Red fluorescence indicated the cell membrane, while green fluorescence indicated the expression of the GFP–CBPA fusion protein. No green fluorescence was detected during early growth; however, it stabilized from T8 onwards (Figure 3A). Figure 3B shows the green fluorescence at T12. Fluorescence observed on the bacteria cell surfaces was indicated by a yellow arrow and on the prespore surface with a red arrow. Consistent with the in silico predictions, GFP fusion experiments revealed that CBPA was located on the cell surface.

### 2.4. Analysis of cbpA Promoter Activity under Alkaline Induction

**Figure 4:**
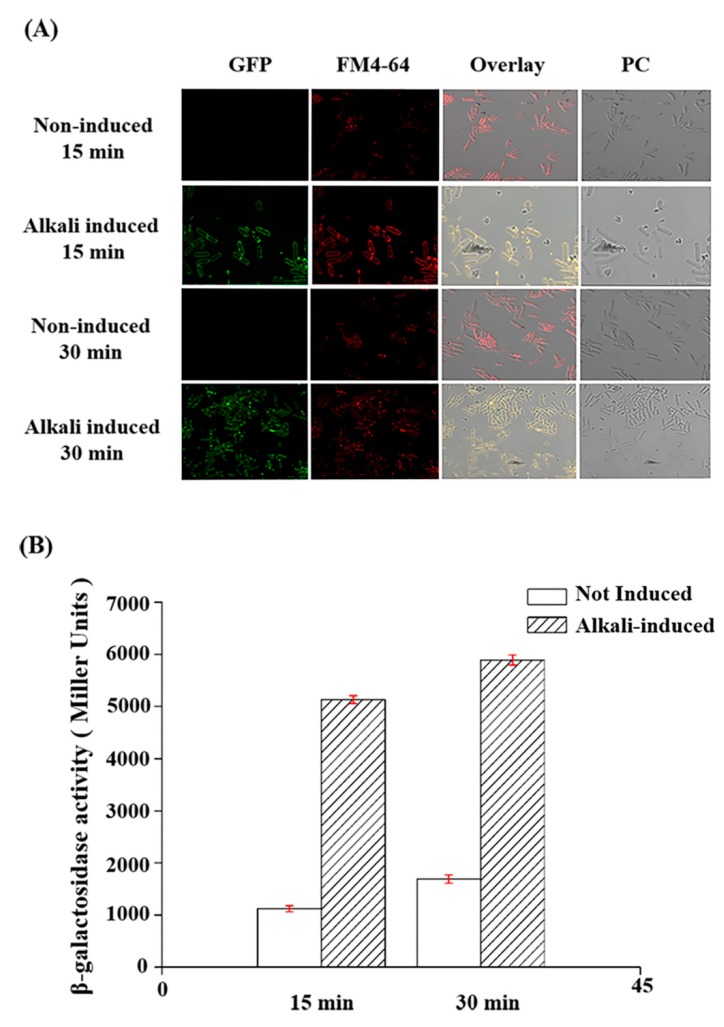
Analysis of transcriptional activity. (**A**) Observation of alkaline induction via laser-scanning confocal fluorescence microscopy. Row 1: non-induced for 15 min. Row 2: induced under alkaline conditions for 15 min. Row 3: non-induced for 30 min. Row 4: induced under alkaline conditions for 30 min. (**B**) Analysis of β-galactosidase activity of the P*cbpA*-*lacZ* fusion +/- alkaline induction.

To investigate the effect of alkali on CBPA expression, we selected an early culture stage wherein the CBPA protein was not expressed. Both the *cbpA-gfp* transduction fusion and the transcriptional activation of the *cbpA* promoter-*lacZ* fusion were analyzed under similar growth conditions. The bacteria were cultured to the late exponential growth stage up to an OD_600 nm_ between 1.5 and 2.0, and an NaOH solution was added to yield a final concentration of 24 mM (pH 9). Samples were maintained under this alkaline environment for 15 and 30 min. GFP expression was then visualized via laser-scanning confocal scanning microscopy (Figure 4A). Cells not treated with the NaOH solution were considered as the negative control. GFP was expressed after alkaline induction (pH 9) but not in the negative control, indicating that CBPA protein expression was rapidly induced under alkaline conditions. In the transcriptional *cbpA* promoter-*lacZ* fusion strain, β-galactosidase activity significantly increased after the addition of NaOH (15 or 30 min) in comparison with non-induced conditions (Figure 4B). Consequently, the cytological observation of the GFP fusion strain and the enzymatic activity analysis in P*cbpA*-*lacZ* promoter fusion strain yielded consistent results, indicating that CBPA expression was induced at an alkaline pH.

### 2.5. Chitin Binding Ability and Chitinase Activity of CBPA

**Figure 5:**
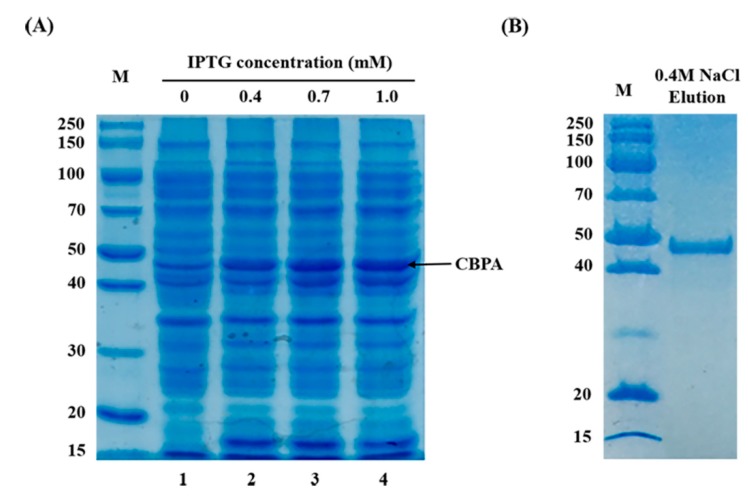
Expression and purification of CBPA proteins harvested for SDS-PAGE analysis. (A) Lane 1: the non-induced expression of CBPA in *E. coli* BL21/DE3. Lanes 2-4: induced expression of HD73-*cbpA* by the isopropyl-β-D-thiogalactopyranoside (IPTG) gradient of 0.4, 0.7 mM, and 1.0 mM. (B) purified CBPA eluted by a gradient of 0.4 M NaCl.

To follow-up on the in silico information indicating CBPA as a chitin binding protein, the next step was to analyze if CBPA really has chitin binding capacity. Therefore, we expressed CBPA as a heterologous recombinant protein. *cbpA* (gene 3189) from Bt HD73 was cloned and expressed in an *E. coli* BL21/DE3 strain. The expected size of the HD73-CBPA protein was 49.78 kDa. Protein expression was induced through an isopropyl-β-D-thiogalactopyranoside (IPTG) gradient, and expression was assessed via SDS-PAGE as a ~50-kDa protein at 0.4–1.0 mM IPTG (Figure 5A). Thereafter, CBPA was purified via chitin affinity chromatography and eluted at a gradient of 0.4 M NaCl, thus showing the chitin-binding capacity of CBPA (Figure 5B). The purified protein band was excised and analyzed via matrix-assisted laser desorption/ionization and time-of-flight peptide mass spectrometry analysis after in-gel digestion, confirming that the heterologous *E. coli*-cloned and -expressed CBPA protein contained the expected peptide composition. Having confirmed its chitin-binding capacity, we assessed for the eventual chitinase activity of the protein. CBPA displayed no chitobiosidase and endochitinase activity (Table 3). Indeed, the fluorescence intensity of chitinase degradation products from various substrates obtained with CBPA approached the same values as those of the negative control. Therefore, CBPA can probably not degrade chitin-rich structures, at least those analyzed herein.

### 2.6. Expression of CBPA-GFP Fusion in Vivo in G. mellonella

**Figure 6:**
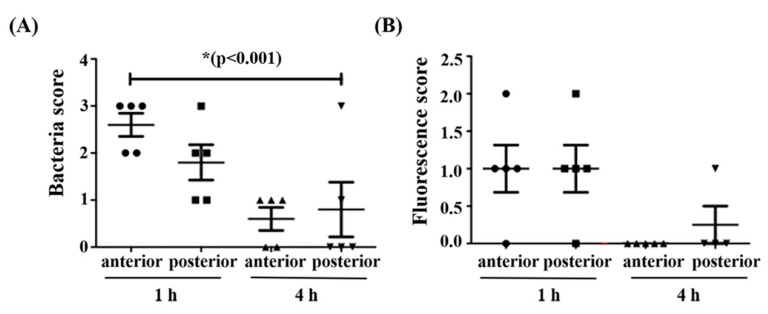
Arbitrary scores of bacteria (**A**) and the expression of the CBPA–GFP fusion protein (**B**) recovered in the *Galleria mellonella* intestine. At one hour and 4 h post-force-feeding with a wild-type HD73 (pHT*cbpA*-*gfp*) strain, samples from the anterior midgut and posterior midgut were analyzed via fluorescence microscopy at 1000× magnification from five chilled, dissected larvae. Scores are as follows: 0 = no bacteria and no fluorescence, 1 = few bacteria <10 per field, 2 = between 10 and 50 bacteria and 3 = more than 50 bacteria per observation field.

Despite the lack of an evident role of CBPA in virulence in the final instar of *Galleria* larvae, we aimed at determining the possibility of CPBA to bind the PM since chitin is a structural element of the PM in all insects. First, we investigated whether CBPA was expressed in the *Galleria* gut, since the aforementioned *in vitro* studies (Figure 3 and Figure 4) indicated that CBPA was expressed on the surface of HD73 cells and under alkaline pH, which may occur in the *Galleria* larval gut. The pH of the *Galleria* midgut was measured via the injection of a liquid pH indicator into three sites of the gut; the pH was between 8.5 and 9 from the anterior to posterior midgut, as directly observed under binoculars with four times magnification. Thereafter, we assessed, via epi-fluorescence microscopy, the presence of fluorescent bacteria from the anterior and posterior midgut of *Galleria* larvae infected with mid log-phase Luria–Bertani (LB) grown vegetative HD73 bacteria carrying the CBPA–GFP plasmid fusion protein. Observations were recorded at 1 and 4 h post-ingestion. The CFU values and the fluorescence intensities were scored upon arbitrary visual observation (Figure 6). Greater CFU values were recovered at 1 than at 4 h post-ingestion (Figure 6A), indicating a relatively rapid intestinal transit and that fluorescent bacteria (Figure 6B) were more abundant at the early time point.

### 2.7. HD73 and HD73 ∆cbpA Intestinal Transit and Localization Assays

Since purified CBPA can bind to chitin, is expressed on the bacterial surface (Figure 3 and Figure 5), and is activated in the gut of *Galleria*, we performed a tight analysis of the persistence of the HD73 wild-type and ∆cbpA mutant strains with vegetative bacteria, presuming that the PM binding capacity of CBPA *in vivo* would lead to a difference between the wild-type and the mutant strains during intestinal transit.

**Figure 7:**
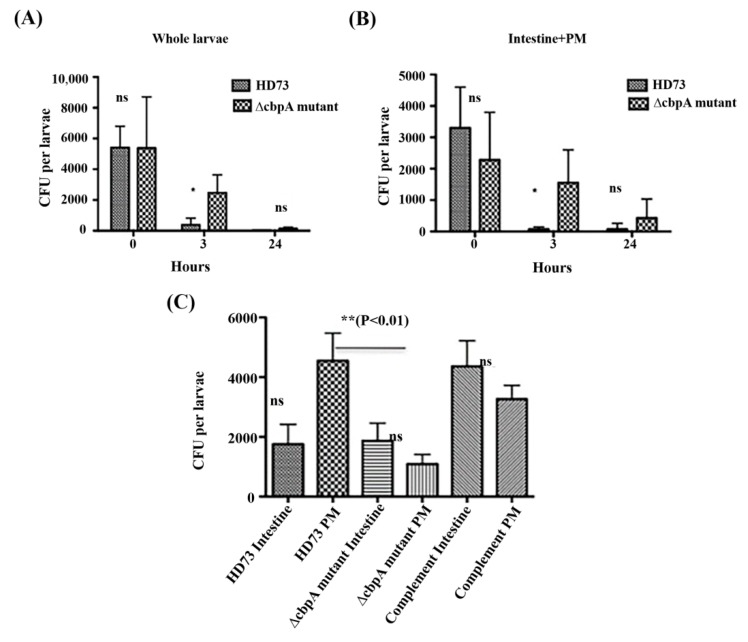
Presence of bacteria in *Galleria mellonella* whole larvae (**A**) dissected complete intestine (**B**), and dissected dissociated intestine and the PM (**C**) from chilled, fifth-instar larvae after force-feeding at stages T0, T3, and T24 h post-feeding for (**A**,**B**) and after 1 h for (**C**). Whole larvae, the larval intestine, and the PM were homogenized to determine the CFU counts for each sample. Assays were repeated at least three times with two replicates per sample time and sample type. CFU counts were analyzed with the PRISM software and one way ANOVA associated with the Bonferroni’s multiple comparison test. ** <0.01 and * <0.05 level, ns (non-significant) different.

First, the presence of bacteria was estimated in whole larvae and dissected whole intestines (gut with the PM) (Figure 7A,B) at three time points. Immediately after ingestion (T0), no difference (≈5000 CFU) was observed between wild-type HD73 and ∆cbpA mutant strains, while at T3 h post-ingestion, a significant difference was observed between wild-type HD73 (≈100 CFU) and the ∆cbpA mutant (approximately 5000 CFU were still observed). At 24 h, no bacteria were observed in larvae fed with wild-type HD73, and approximately 100 CFU were recorded for larvae infected with the ∆cbpA mutant. A similar analysis was performed with the dissected whole intestines (gut and the PM) (Figure 7B). No difference was observed at T0; however, at T3 h post-ingestion, almost no bacteria were observed with the wild-type HD73 strain, while approximately 3000 CFU were still observed with the ∆cbpA mutant strain. Thus, bacteria not expressing CBPA persist longer in the gut than the wild-type bacteria, suggesting that the wild-type HD73 bacteria are more easily excreted with the PM during natural food bolus transit, resulting in feces production. The feces are surrounded by the PM [6].

Therefore, we further analyzed the speed of transit after force-feeding with spores in order to uncover the time where we would still find all bacteria in the insect before they would be excreted with the feces. Feces were collected and assessed for the presence of bacteria, and the mean numbers of feces per larvae were recorded at four time points. Feces were observed at 2 h (0.3 feces/larvae) and displayed an increase in the mean number of feces at 3 and 4 h to 1.5 feces per larva. The presence of the bacteria was found in feces from the 2 to early 3 h post ingestion, showing that the mean transit time under these conditions was approximately 2 h. Based on these observations, we thereafter tested for the presence of the bacteria adhering to the PM. Hence, we selected the time point of 1 h post-ingestion, since feces were excreted at 2 h per the aforementioned results and since at 3 h post-ingestion, only a few residual bacteria were observed in the wild-type HD73-treated larvae (Figure 7A,B). The CFU value recovered from the dissected PM alone (Figure 7C) was approximately 5000 for wild-type HD73 and three-fold lesser for the ∆cbpA mutant, which was significantly different from the wild-type. In addition, the *cbpA*-complemented ∆cbpA mutant recovered a better adhesion to the PM. One way ANOVA analysis and the Bonferroni’s multiple comparison test showed significant differences between the PM from the ∆cbpA mutant and wild-type HD73, while the complemented strain PM had no significant differences with the others. The observed variations in CFU that were associated with the dissected intestine, separated intestine, and the PM may have been due to the difficulty of the dissection approach (see Materials and Methods). The results indicated that CBPA *in vivo* has an affinity for the PM (Figure 7C) and therefore may help retain the bacteria to this tissue, which could then increase the infection efficacy of Bt.

## 3. Discussion

During infection, a pathogen interacts with the host and circumvents the host’s defense mechanisms. For many pathogens, the first step of colonization depends on the capacity to adhere to the host tissue via multiple factors [5]. Therefore, pathogenic bacteria produce surface molecules and appendages, such as flagella and pili. They sense host surfaces, thus facilitating their adhesion with host cells and thereby bringing secreted molecules close to the host cell targets. Pore-forming Cry endotoxins are the major *B. thuringiensis* insecticidal effectors. They bind to specific membrane receptors on the larval midgut epithelium [1,33]. Meanwhile, the spores and their outgrown vegetative form participate in infection [3]. Several proteases, lipases, chitinases, toxins, or adaptation factors are involved in pathogenesis [8,31] and in fulfilling the insect phase of the Bt life cycle. The germination and growth of *B. thuringiensis* in the gut of insect larvae have been previously photographically studied. It was reported that the spores of *B. thuringiensis* germinated at the surface of the epithelium 40–120 min after inoculation [34]. Additionally, histological studies have shown that the vegetative bacteria of *B. thuringiensis* are found in the gut lumen of *Chrysomela* [35]. However, thus far, limited information is available regarding the role of factors associated with Bt spores and newly outgrown vegetative bacteria in the very early stages of gut infection, particularly with respect to their interaction with the PM.

This study investigated the expression and functions of a yet unknown chitin-binding protein (CBPA) from the Bt HD73 strain and explored its role in insect infection. The gene encoding CBPA was previously identified among genes activated under alkaline conditions via an *in vitro* transcriptomic screening [28], and our previous *in silico* analysis indicated its putative chitin-binding function and its presence in several *B. cereus* genomes. Herein, we investigated the spatiotemporal aspects of its expression. The confocal microscopic imaging of Bt HD73 harboring a CBPA–GFP fusion protein revealed that CBPA was expressed in vitro at the late stationary growth stage and localized on both the bacterial and prespore surfaces (Figure 3). Furthermore, when the bacteria were exposed to an alkaline pH, the protein was expressed as soon as 15 min post-induction, as revealed through both CBPA–GFP fusion and a lacZ transcriptional promoter fusion (Figure 4). These observations indicate that expression is inducible at an alkaline pH, which is known for the Lepidopteran midgut environment. This has also been reported in the case of the Bt CBP-21 chitin-binding protein [36] and is concurrent with our former transcriptome findings [28] and with the present study, wherein the CBPA–GFP protein was observed in the *Galleria* midgut at 1 h post-ingestion with vegetative bacteria.

The widespread presence of CBP proteins in bacteria and other organisms implies their importance, with chitin binding being the most common function. CBP21 from the Bt HD1 strain binds chitin in insects [36], and CBP50 from the Bt *konkukian* serotype and CBP33A from *Lactococcus lactis* bind insoluble chitin (α and β), colloidal chitin, and cellulose [37]. Furthermore, *Streptomyces* can secrete small proteins that specifically bind α-chitin [38]. ChbB produced by *Bacillus amyloliquefaciens* preferentially binds β-chitin [39]. The present study showed the capacity of recombinant purified CBPA to bind chitin, which is concurrent with previous reports with similar CBP proteins.

Some bacteria simultaneously produce CBP and chitinase, thus improving the hydrolysis efficiency of chitinases. For example, CBP24 and CBP50 produced by Bt *serovar konkukian* act synergistically with bacterial chitinases for chitin degradation [40,41], CBP21 from *Serratia marcescens* exerts a synergistic effect with chitinase on its hydrolysis efficiency [19], and CBP33A from *Lactococcus lactis* can increase the hydrolysis efficiency of chitinase Chi18A [20]. As expected, chitinase activity was not observed for CBPA itself, but, based on our findings, we may suggest that CBPA on the bacterial surface can help target the bacteria to the chitin-rich PM, where the chitinases are of particular relevance. Indeed, the present results showed that the ∆cbpA mutant adhered less well to the *Galleria mellonella* PM, thus indicating that CBPA is involved in the adhesion of Bt HD73 out-grown vegetative bacteria to the PM (Figure 7C).

CBPA localized on the cell surfaces in a manner similar to CBP21 from the Bt HD1 strain, which was also reported to be present in the spore crystal preparation [36]. CBP21 and CBPA have low global sequence homology, and an *in silico* analysis indicated that CBPA comprises the chitin-binding-3 domain, two FN3 domains, and a CBM-5-12 domain, while CBP21 comprises a peptidase M73 domain. It was speculated that Bt-CBP21 interacts with Cry1Ac to potentiate its insecticidal activity [36], which may be concurrent with an earlier observation with strain Bt HD73, wherein Cry1Ac localized at the spore surface [42]. In the present study, Cry1Ac and spores from ΔcbpA mutant displayed higher LC_50_ (approximately 10-fold) values against *Ostrinia furnacalis* (Asian Corn borer) neonate larvae in comparison with the wild-type Bt HD73 strain, and the *cbpA*-complemented ΔcbpA mutant strain displayed a similar LC_50_ value to the wild-type strain, indicating the role of CBPA in virulence in the HD73 strain. As the first barrier in the digestive tube in most insects is the PM, orally acting pathogens require factors that can interfere with the PM. In the present study, CBPA increased the adherence to the PM, thus playing a role in the early stage of infection. In the susceptible insect, the Asian corn borer, the absence of CBPA strongly reduced mortality, which was not the case for *Galleria,* where no mortality was recorded with the wild-type HD73 strain or ∆cbpA mutant strain alone. Therefore, under the present conditions, no obvious function of CBPA in virulence was discerned in *Galleria*, probably because the strong synergism with Cry1Ca [31] concealed a rather subtle bacterial effect or because Cry1Ca is acting directly on the PM, consequently reducing the role of CBPA in that synergy model. However, the *Galleria* model was optimal to assess bacteria–PM interactions *in vivo*. Indeed, the transit studies in *Galleria* clearly indicated that CBPA plays a role *in vivo*, as its presence increases the capacity of vegetative bacteria to adhere to the PM.

**Figure 8:**
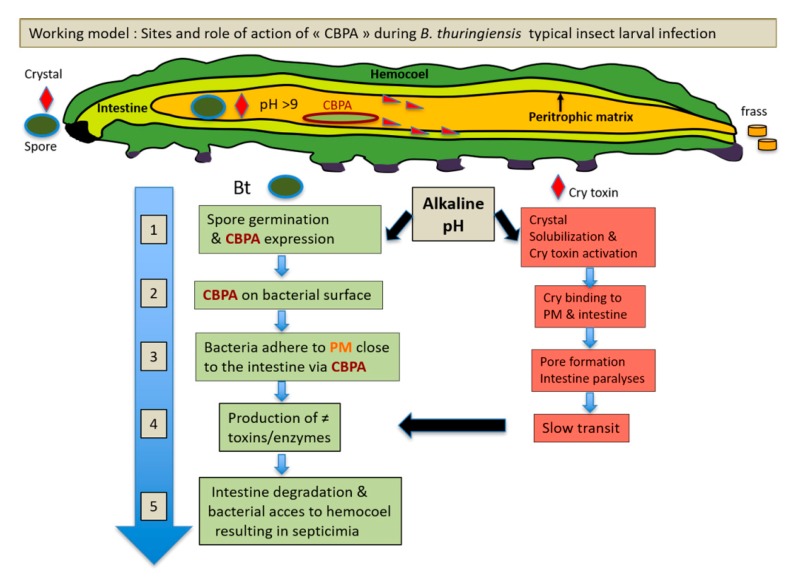
Proposed working model for the site of action of the chitin binding protein CBPA. The figure indicates where CBPA, during the oral infection with *B. thuringiensis* in a Cry toxin susceptible lepidopteron larva, plays a role. The green blocks of the steps refer to the spore/bacteria actions, and the red blocks refer to the role of the Cry toxins. The numbers, in the time scale arrow (in blue), indicates the order of events of which some occurs simultaneously. Our results showed that CBPA is expressed on the surface of vegetative bacteria (step 1) and is induced at alkaline pH. The proposed major role of CBPA is its adhesion to the peritrophic matrix (PM) (steps 1 and 3), which permits outgrown bacteria to bind to the PM and to be closer to the intestinal surface (4 and 5) in order to facilitate the tissue damaging action of secreted enzymes and toxins, e.g., from the PlcR regulon [31].

Concurrent with previous reports, the present study proposes an additional step in the mode of action of *B. thuringiensis* (Figure 8). The present results indicated that CBPA can be induced in vegetative Bt cells in the alkaline midgut environment, thus facilitating the adhesion of Bt bacteria to the PM and thereby increasing the performance of various virulence factors. Accordingly, chitinases or enhancin-like proteins (Bel and MpbE) [43,44] may be produced and destabilize the chitin structure of the PM. This, along with the role of the active pore-forming Cry toxins known to damage the midgut cells resulting in reduced PM renewal and reduced intestinal transit time, further facilitates bacterial adhesion with intestinal cells and increases colonization. This might be followed up by bacterial translocation from the midgut to the insect hemocoel, through the action of non-specific adaptation and virulence factors, notably those from the PlcR regulon, which was earlier shown to being important for virulence toward *Galleria* [31]. Therefore, the present results further the current understanding of the complex pathogenesis and ecology of Bt, owing to studies on two insects: *Ostrina*, which is naturally sensitive to Bt HD73 and its Cry1Ac toxin, and the non-sensitive model insect *Galleria*, which allows for the easy manipulation of the PM. Further studies are required to analyze the importance of CBPA in other Bt strains and insects in order to understand the mode of action of CBPA and to validate our findings as a general feature in the early stages of Bt insect larva infection.

## 4. Materials and Methods

### 4.1. Bacterial Strains

The plasmids, primers, and sequences used herein are enlisted in Table 4 and Table 5. Bt strains were cultured at 30 °C, and *Escherichia coli* was cultured at 37 °C with agitation at 220 rpm in an LB medium (1% NaCl, 1% tryptone, and 0.5% yeast extract) [45]. *B. thuringiensis* HD73 (the wild-type strain, producing crystals exclusively comprising the Cry1Ac toxin) was used to clone the target gene and monitor promoter activity as the recipient strain [46], as well as for bioassays and mutant construction. DNA sequences were obtained from the NCBI database (https://www.ncbi.nlm.nih.gov/) and compared using the Basic Local Alignment Search Tool (BLAST).

### 4.2. Insects

For *O. furnacalis* (Asian corn borer), larvae for bioassays and mortality tests (see below), were provided by the rearing at the Chinese Academy of Agriculture Sciences (Beijing, China). For mortality tests and other in vivo analyses, 5th instar larvae of the greater wax moth *G. mellonella* were used. Insects were reared at the INRAE-Micalis, Jouy en Josas, France, facilities at 27 °C and fed with pollen and bee wax (La ruche Roannaise, France). Prior to assays, the larvae weighting approximately 250 mg were selected and stored under starvation conditions for 24 h.

### 4.3. DNA Manipulation and Transformation

PCR amplifications were performed using Taq DNA polymerase and Pfu DNA polymerase (TIANGEN Biotechnologies Corporation, Beijing, China). PCR products were separated on agarose gels and recovered using the HiPure Gel Pure DNA Mini Kit (Magen Biotechnology Corporation, Guangzhou, China), and plasmid DNA was extracted from *E. coli* using the Plasmid Miniprep Kit (Axygen Biotechnology Corporation, Hangzhou, China) in accordance with the manufacturers’ instructions. Restriction enzymes and T4 DNA ligase (Thermo Fisher Scientific, Beijing, China) were used in accordance with the manufacturer’s instructions. Oligo-nucleotide primers were synthesized by Sangon Biotech (Shanghai, China). All constructs were confirmed by DNA sequencing (GENEWIZ, Beijing, China). *E. coli* cells were transformed via standard procedures [53], and Bt cells were transformed via electroporation, as described previously [54].

### 4.4. Cloning of the HD73-cbpA Gene

The *HD73-cbpA* gene (ID:14557228) was cloned from the wild-type HD73 genome via PCR, using specific primers *cbpA*-a and *cbpA*-b (Table 5) under the following cycling conditions: denaturation at 94 °C for 30 s, annealing at 50 °C for 1 min, and extension at 72 °C for 1 min for 34 cycles. The size of the PCR products was 1368 bp, which was digested with *Bam*H I and *Sal* I. Thereafter, the fragment of the *cbpA* gene was inserted into the expression vector pET21b (Novagen, Bloemfontein, South Africa) and digested with the aforementioned restriction enzymes. The recombinant plasmid was transformed into *E. coli* JM110 for amplification and preservation. The recombinant plasmid was selected using Amp^r^ on the vector to select transformants and to obtain potentially positive clones via PCR, followed by NCBI BLAST to verify the correct sequence. Finally, the recombinant plasmid was transformed into *E. coli* BL21/DE3 for expression.

### 4.5. Expression and Purification of CBPA

*E. coli* BL21-harboring pET*cbpA* were cultured in an LB medium up to the logarithmic phase (A_600_ = 0.8 to 1.0), and the culture was cooled to 20 °C and induced with IPTG at a step-down gradient of the final concentration from 0.4 to 1.0 mM at 150 rpm for 20 h. The cells were harvested via centrifugation (6000 rpm/min, 10 min, and 4 °C) and resuspended in a 20 mM Tris-HCl buffer (pH = 8.0). Thereafter, the supernatant (cytosol) and pellet of the crude protein extract were obtained via centrifugation (12,000 rpm/min, 20 min, and 4 °C), followed by bacterial cell lysis using an ultrasonic cell disruption system. Protein expression was analyzed via SDS-PAGE (10% resolving gel). The protein was incubated with chitin beads, and the bound protein was purified after elution with an NaCl solution containing a step-up gradient from 0 to 1.0 M.

### 4.6. Determination of Chitinase Activity

The chitinase activity of the CBPA protein was detected using the Sigma-Aldrich Chitinase Assay Fluorimetric kit (Sigma-Aldrich) in accordance with the manufacturer’s instructions. First, the 4-MU standard solution was prepared, and fluorescence was measured. Thereafter, the CBPA protein (1.21 mg/mL) was added to three different substrates. Green Trichoderma chitinase was used as a positive control. The substrate reaction solution and the standard solution were equilibrated in a 37 °C water bath for 5–10 min. The standard sample and the reaction sample were prepared (10 µL) in accordance with the manufacturer’s instructions before being subjected to agitation in parallel. The sample was incubated in a 37 °C warm bath for 30–60 min. Finally, 200 µL of a stop solution was added, and fluorescence was measured at an excitation wavelength of 360 nm and an emission wavelength of 450 nm. The concentration of the target solution was determined from a standard curve with chitinase as the positive control.

### 4.7. Construction and Expression of Recombinant gfp-conjugated cbpA

Both the 1379-bp fragment of the *cbpA* ORF and the 541-bp upstream sequence comprising the promoter were amplified via PCR with the specific primers *gfp*-1 and *gfp*-2 (Table 5), using the HD73 genome as the template. A 48-bp linker fragment (TCAGGTGGAGGCGGTTCAGGCGGAGGTGGCTCTGGCGGTGGCGGATCG) and a 717-bp GFP ORF were amplified via PCR with specific primers *gfp*-3 and *gfp*-4 (Table 5), using the Cry1Ac-GFP plasmid as the template [55]. The fusion fragment was amplified via overlapping PCR and inserted into the shuttle vector pHT315, as described previously [49], using the *Sph*I and *Bam*HI restriction sites. Thereafter, the recombinant plasmid was transformed into HD73 via electroporation.

### 4.8. Laser-Scanning Confocal Microscopy of CBPA-GFP Fusions

A single colony was inoculated in an LB medium, cultured overnight at 30 °C with agitation at 220 rpm, and 1% of the inoculum was seeded in 100 mL of the LB medium and incubated until the OD_600_ value approached 2.0–2.2, which is the end point of the exponential phage (T0) according to the previously established growth curve. One-milliliter bacterial aliquots were taken every 1 h for centrifugation to obtain the precipitation (30 °C, 12,000 rpm for 1 min) and washed twice with deionized water (200 µL). Thereafter, bacterial cells were resuspended in a specific amount of deionized water. Different samples were analyzed at time points T1, T2, etc., (Tn means n hours after T0 entrance into stationary phase). A red fluorescent membrane stain FM4-64 (Molecular Probes, Inc., Eugene, OR, USA) was suspended in dimethyl sulfoxide to a final concentration of 100 µM and incubated on ice for 1 min. Five-hundred nanoliters of the bacterial sample and an equal volume of FM4-64 were mixed and placed on a glass slide, covered with a coverslip, and sealed with a transparent nail polish. FM4-64-stained bacteria were observed using a 63× oil-immersion lens and scanned using a laser-scanning confocal microscope (Leica TCS SL; Leica Microsystems, Wetzlar, Germany). The FM4-64 was detected at an excitation wavelength of 514–543 nm; GFP was detected at 633 nm.

### 4.9. Construction of the Transcriptional Promoter P*_cbpA_-lacZ* Fusion Gene

The sequence upstream from the *cbpA* gene, where the promoter fragment of P*_3189_* is located, was cloned using the specific primers P*_cbpA_*-F and P*_cbpA_*-R (Table 5) from the wild-type HD73 genome, under the following cycling conditions: denaturation at 95 °C for 20 s, annealing at 50 °C for 20 s and extension at 72 °C for 1 min for 30 cycles. The fragment of the P*_cbpA_* promoter was inserted into vector pHT304-18Z using the *Pst*I and *Bam*HI restriction sites. The vector pHT304-18Z harbored a promoter-less *lacZ* gene [50]. Thereafter, the recombinant plasmid pHTP*_cbpA_* was transformed into HD73 via electroporation, and positive strains were selected on the basis of the Erm^r^ phenotype and via PCR identification.

### 4.10. β-Galactosidase Assays

Bt strains containing *lacZ* fusion transcripts were cultured in LB at 30 °C and at 220 rpm with appropriate antibiotics up to an OD_600_ value of 1.5–2.0. Thereafter, NaOH was added to a final concentration of 24 mM (pH 9). No NaOH was added to the control culture. Two-milliliter aliquots were harvested from the experimental and control cultures at 15 and 30 min, and β-galactosidase activity (Miller units per milligram of protein) of the cell pellets was measured as described previously [56]. Final values were determined using the data processing software Original 8.0 and SPSS.

### 4.11. Construction of the HD-73 ∆cbpA Mutant

The interruption mutant was obtained via homologous recombination and insertion-replacement with a kanamycin resistance-encoding DNA cassette. The upstream gene fragment *cbpA*-u (317 bp) was obtained with the use of specific primers *cbpA*-A and *cbpA*-B and the downstream gene fragment 3189-d (504 bp) using primers *cbpA*-C and *cbpA*-D, with the genomic DNA of wild-type strain HD73 as the template. The kanamycin resistance gene cassette was a 1495-bp fragment. Primers *cbpA*-A and *cbpA*-D were used to ligate the aforementioned three fragments via overlapping PCR. Thereafter, the cassette fragment (2243 bp) was inserted into the temperature-sensitive suicide mutant erythromycin-resistant plasmid pRN5101 [51] at the *Bam*HI and *Sal*I restriction sites, finally yielding the pRN5101Ω*cbpA* plasmid. Positive transformant mutants were selected as reported previously [48].

### 4.12. Complementation of the HD-73 ∆cbpA Mutant

Oligonucleotide primers *cbpA*-c (with a pHT 304 upstream homologous arm) and *cbpA*-d (with a pHT 304 downstream homologous arm) (Table 5) were used to amplify the *cbpA* gene and its own promoter by using HD73 genomic DNA as the template. The amplified fragment (1946 bp) was ligated into the shuttle vector pHT304 to generate pHTC*cbpA* using a recombinant enzyme. The resulting plasmid (pHTC*cbpA*) was amplified in *E. coli* and introduced into the HD-73 ∆cbpA mutant strain. Genetically-complemented mutant HD73 strains (Δ*cbpA*::*cbpA*) were obtained by transforming pHTC*cbpA* into HD73Δ*cbpA* cells.

### 4.13. Crystal Spore Mixture Preparation for Asian Corn Borer Bioassays

A single colony of wild-type and mutant strains of Bt HD73 was inoculated in 20 mL of an LB liquid medium and cultured overnight at 30 °C with agitation. The activated bacteria were then transferred to 300 mL of an LB broth at a ratio of 1% and cultured at 180 rpm for 4–5 h to the logarithmic growth phase. Thereafter, the bacterial culture supernatant was transferred to 300 mL of an LP beef extract peptone medium at a ratio of 1% for approximately 40 h at 30°C, and the crystal cleavage rate was observed to be greater than 50%. The crystals, spores, and debris were harvested via centrifugation at 6000 rpm for 20 min (20 °C). The pellet was washed with water and centrifuged at 6000 rpm for 20 min (20 °C), and the supernatant was discarded. The pellet was resuspended in deionized water to obtain the final crystal and spore suspension.

### 4.14. Spore Preparation for Asian Corn Borer Bioassays

A single colony of the HD73, ΔcbpA mutant, and the complemented strains was inoculated in 20 mL of the LB broth and cultured overnight at 30 °C with agitation. Activated bacteria were transferred to 300 mL of a CCY liquid medium (MgCl_2_·6H_2_O: 0.5 mmol/L, MnCl_2_·4H_2_O: 0.01 mmol/L, FeCl_3_·6H_2_O: 0.05 mmol/L, ZnCl_2_: 0.05 mmol/L, CaCl_2_.6H_2_O: 0.2 mmol/L, KH_2_PO_4_: 13 mmol/L, K_2_HPO_4_: 26 mmol/L, L-Glutamine: 20 mg/L, Casamino acids hydrolysate: 1 g/L, Yeast extract: 0.4 g/L, glycerol: 0.6g/L) based on a ratio of 1% and cultured at 220 rpm for 72 h up to a spore percentage of >99%, as reported previously [57]. The spores were harvested via centrifugation at 6000 rpm for 10 min (4 °C). The pellet was washed with water and centrifuged at 6000 rpm for 10 min, and the supernatant was discarded. The pellet was resuspended in deionized water to obtain a spore suspension.

### 4.15. Spore Counts

Spore suspensions of the wild-type HD73, ΔcbpA mutant, and the complemented strains were administered a heat treatment (65 °C for 30 min) to eliminate all vegetative cells. Thereafter, 100 µL of the 10^−6^ dilutions of each sample were plated on the LB agar medium and incubated at 30 °C for 12 h. The colony characteristics of the Bt culture were assessed as described previously [58].

### 4.16. Dose-Mortality Response Bioassays Against Asian Corn Borer

SDS-PAGE was performed to analyze protein profiles and concentrations, using bovine serum albumin (BSA) as the standard. Bioassays were performed as described previously [59]. Cry1Ac was prepared as described previously [60]. Insecticidal activity against the Asian corn borer, *O. furnacalis* (Guenée) was assayed by administering neonates with an artificial diet supplemented with a 0.01 µg Cry1Ac/g diet and different concentrations of spores (10^4^–10^8^ CFU/g diet) prepared from the HD73 wild-type, ΔcbpA mutant, and the complemented strains. The spores were heat-treated (65 °C for 30 min) to eliminate all vegetative cells and crystals. The feed was uniformly distributed into 48-well trays, each well containing approximately 200 mg of feed and infested with one neonate larva. Assays were carried out at 27 ± 1 °C with a L16/D 8-h photoperiod and 70–80% relative humidity. Survivors were recorded after 7 d. Deionized water was used as the negative control. Each assay was performed in triplicate.

### 4.17. In Vivo G. mellonella Virulence Assays with HD73 and ∆cbpA Mutant Strains

Oral force-feeding assays were performed as described previously [32,61]. A dose of 3–5 × 10^6^ spores or mid-log LB grown bacteria from HD-73 wild-type and ∆cbpA mutants was suspended in 10 µL of 0.3 mg/mL Cry1C activated toxin or in 1% NaCl water (negative control) using a needle and syringe for the accurate distribution to each larvae. At least 20 larvae per condition were incubated at 37 °C under starvation conditions, and mortality was scored at 24 h post-infection. Experiments were performed in triplicate. The inocula were evaluated via plating after serial dilution.

### 4.18. Expression of the CBPA-GFP Fusion Protein in Vivo in G. mellonella

CBPA expression during intestinal transit was scored via the fluorescence microscopic examination of intestinal samples from the anterior and posterior midgut of larvae infected with HD73 (pHT-*cbpA-gfp*). At least 5 larvae were dissected, and gut samples were observed using the Fluorescence Zeiss-Observer microscope with a 100× oil-immersion objective lens with a GFP filter at 1 and 3 h post-ingestion. CFU counts and fluorescence levels were scored through arbitrary visual observation, since only few bacteria were present, and the fluorescence intensity was too low for imaging or fluorescence-activated cell sorting analysis.

### 4.19. Intestinal Particle Transit Time for Final-Instar G. mellonella

Final-instar larvae starved for 24 h, similar to those in the virulence assays, were used. The transit time was recorded by observing the first appearance of feces and the presence of bacteria in the feces. Thirty larvae were force-fed with the same dose of spores as in the virulence assay. Larvae were placed individually in a 24-well micro-titer plate. Feces were harvested and enumerated at 1, 3, 4, and 24 h after force-feeding. The CFU value was evaluated in the feces at 1 and 2 h post-ingestion via the plating of 200 µL suspension in 1% NaCl water.

### 4.20. HD73 and HD-73 ∆cbpA Intestinal Transit and Localization Assays

To analyze the effect of *cbpA* deletion on bacterial transit following oral infection, a condition devoid of the Cry1C toxin was used. In total, 3–5 × 10^6^ mid-log-phase bacteria were force-fed to 5^th^ instar *G. mellonella* larvae. CFU values were recorded after plating at different time points and with four different larval samples: whole larvae, dissected whole intestine, intestine without the PM, and the PM alone. First, alcohol (70%, 1 min)-cleaned larvae were homogenized in a PBS (phosphate buffed saline pH 7.4) buffer using an Ultraturax mixer at the rate of 2 larvae per 4 mL. Two completely dissected intestines were homogenized with Ultraturax in 1.5 mL 1% NaCl water. CFU values per larva were recorded at 0, 3, and 24 h post-feeding in at least 2 larvae and repeated 3–4 times. To record bacteria adhering to the PM, larvae were incubated on ice 1 h post-ingestion and gently dissected with the help of chirurgical scissors and tweezers to obtain the PM and intestine alone. The cooled larvae were placed on the dorsal under the binoculars and gently opened with the scissors from the rectum to the head, and the skin is maintained with needles. Fat body, silk glands, and other tissue were gently moved to only expose the digestive tube (DT) (the whole intestine from foregut to hindgut). The DT was cut at two sites, one just above the foregut and one above the rectum, and moved to a clean glass slide. Then, a small cut with the scissors was performed just above the hindgut, and the PM was gently pooled out and separated from the intestine with the fine tweezers; the PM and the intestine were placed directly in the respective tubes prior to homogenization. Two PMs were crushed with a small pestle and 5–6 glass beads in 200 µL 1% NaCl water, and an additional 200 µL of NaCl were added before serial dilution and plating. This experiment was performed in triplicate for each strain. The intestines alone were processed as for the above whole intestines method.

## Figures and Tables

**Table 1:** Mortality of crystal and spore mixture against Asian corn borer larvae.

No.	Concentration of Cry Protein (μg/g)	Number of Spore (Numbers/g)	Mortality (%)		Significance
			Wild-Type	∆cbpA Mutant	
1	0.005	5.4 × 10^6^	0.0 ± 0.0	0.7 ± 0.7	0.374
2	0.025	2.7 × 10^7^	4.7 ± 0.7	2.7 ± 1.3	0.251
3	0.050	5.4 × 10^7^	17.0 ± 1.2	8.7 ± 0.7*	0.003
4	0.250	2.7 × 10^8^	35.3 ± 1.5	19.7 ± 0.7 *	0.001
5	0.500	5.4 × 10^8^	51.3 ± 1.8	33.7 ± 1.3*	0.001
6	1.000	1.1 × 10^9^	76.3 ± 1.3	54.7 ± 0.7*	<< 0.001**
7	2.500	2.7 × 10^9^	90.3 ± 2.7	58.7 ± 1.3 *	<<0.001***

* Means within a line were significantly different (*p* ≤ 0.01) via the t-test. ** significance: *p* = 0.000130. *** significance: *p* = 0.000478.

**Table 2:** LC_50_ values of different strains against Asian corn borer.

	LC_50_(Spore CFU)	95% Confidence Interval
BtHD73	6.59 × 10^5^	3.41 × 10^5^–1.04 × 10^6^
△cbpA mutant	4.85 × 10^6^	2.25 × 10^6^–7.61 × 10^6^
△cbpA*::cbpA*	5.72 × 10^5^	3.18 × 10^5^–8.98 × 10^5^

Each concentration of spore was mixed with Cry1Ac at a final concentration of 0.01 ug/g.

**Table 3:** Fluorescence value of chitinase activity.

Chitinase Substrate	Chitinase Activity (Substrate Degradation, μmol/min)		
	Negative Control	CPBA	Positive Control
4-Methylumbelliferyl N-acetyl-β-D-glucosaminide	4.79 × 10^4^	4.88 ×1 0^4^	3.47 × 10^6^
4-Methylumbelliferyl Β-D-N,N’-diacetylchitobioside hydrate	6.35 × 10^4^	5.88 × 10^4^	4.81 × 10^6^
4-Methylumbelliferyl Β-D-N,N,N″-triacetylchitotriose	5.01 × 10^4^	4.79 × 10^4^	2.21 × 10^6^

**Table 4:** Strains and plasmids used in this study.

Strain or Plasmid	Characeristics	Reference or Source
*E. coli* strains		
JM110	*rpsL(str^r^),thr,leu,thi-1,lacY,galK,galT,ara,tonA,tsx,dam,dcm,supE44,Δ(lac-proAB),* *[F’,traD36,proAB,laclqZΔM15]*	This laboratory
BL21/DE3	*E. coli* B, *F-,dcm,ompT*, *hsdS(rB-mB-), gal,* *λ(DE3)*	[47]
ET	*∆(lac-proAB) RpsL(str^r^), thr, leu, endA, thi-1, lacY, galK, galT, ara, tonA, tsx, dam, dcm, supE44, (F’ traD36proABlacI^q^Z∆M15)*	This laboratory
***B. thuringiensis* subsp. *kurstaki* strains**		
HD73	Contains *cry1Ac* gene	[46]
HD73(pHT-*gfp*)	HD73 strain containing plasmid pHT-*gfp*	[48]
HD73(pHT-*cbpA-gfp*)	HD73 strain containing the translational fusion plasmid pHT*-cbpA-gfp*	This study
HD (P*_cbpA_-lacZ*)	HD73 strain containing plasmid pHTP*_cbpA_*	This study
HD73(pRN5101Ω*cbpA*)	HD73 strain containing plasmid pRN5101Ω*cbpA*	This study
HD73(ΔcbpA)	HD73 mutant, Δ*cbpA*	This study
HD73(Δ*cbpA*::*cbpA*)	HD73(Δ*cbpA)* containing plasmid pHTC*cbpA*	This study
***Plasmids***		
pET-21b	Expression vector, Amp^r^, 5.4 kb	Novagen
BL21 (pET*cbpA*)	BL21(DE3) with pET*cbpA* plasmid	This study
pHT315	*B. thuringiensis-E. coli* shuttle vector, 6.5kb	[49]
pHT304-18Z	Promoterless *lacZ* vector, Ery^r^ Amp^r^, 9.7 kb	[50]
pHTP*_cbpA_*	pHT304-18Z carrying *P_cbpA,_* Amp^r^ Erm^r^	This study
pET*cbpA*	pET-21b containing *cbpA* gene, Amp^r^	This study
pETC*cbpA*	pHT304 carrying *cbpA* gene,Amp^r^	This study
pHT P*_cbpA_-3189-gfp*	pHT315 containing P*_cbpA_-cbpA-gfp* gene	This study
pRN5101	Temperature-sensitive plasmid, 8.0 kb	[51]
pRN5101Ω*cbpA*	pRN5101 carrying partial *cbpA* deletion gene	This study
pDG780	Containing a kanamycin resistance gene	[52]

**Table 5:** Primers and sequences used in this study.

Primer	Sequence	Restriction Site
*cbpA*-a	CGCGGATCCGATGAACATGAATAATCGAT	*Bam*H I
*cbpA*-b	ACGCGTCGACTTACACTGTTTTCCATAAT	*Sal* I
*cbpA*-c	GCATGCCTGCAGGTCGACTCTAGAGGGCTGCTTTGAATTTGAAGGAAT	
*cbpA-d*	TGTAAAACGACGGCCAGTGAATTAAAGCCCATCATCTCTTAGTTCAT	
*gfp*-1	CGGGATCCAAGAGGCTGCTTTGAATTTGAAGG	*Bam*H I
*gfp*-2	CCGCCTCCACCTGACACTGTTTTCCATAATG	
*gfp*-3	CATTATGGAAAACAGTGTCAGGTGGAGGCGG	
*gfp*-4	CATGCATGCTTATTTGTATAGTTCATCCATGCC	*Sph*I(*Pae* I)
P*_cbpA_*-F	TGCACTGCAGGGCTGCTTTGAATTTGAAGGAATC	*Pst* I
P*_cbpA_*-R	CGGGATCCGTTCATGTCCCCTTCTTGTTATAC	*Bam*H I

Underline indicates the restriction enzyme site.

## Supplementary Materials

The following are available online at https://www.mdpi.com/2072-6651/12/4/252/s1, Figure S1: Analysis of mortality of *Galleria mellonella*.
