# Supplementary material for: A Bacillus thuringiensis Chitin-Binding Protein is Involved in Insect Peritrophic Matrix Adhesion and Takes Part in the Infection Process
Source: Toxins (Basel). 2020 Apr 13;12(4):252. doi: 10.3390/toxins12040252 (PMC7232397; doi:10.3390/toxins12040252)
Supplement: Supplementary file 1 [file toxins-12-00252-s001.pdf]

Supplementary materials: A *Bacillus thuringiensis* Chitin-Binding Protein is Involved in Insect Peritrophic Matrix Adhesion, and Takes Part in the Infection Process

Jiaxin Qin, Zongxing Tong, Yiling Zhan, Christophe Buisson, Fuping Song, Kanglai He, Christina Nielsen-LeRoux and Shuyuan Guo

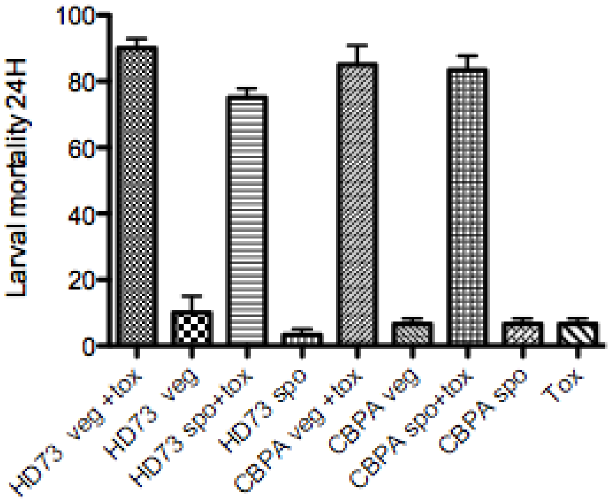

Figure S1. Analysis of mortality of *Galleria mellonella.* Larvae were force-fed with 5 × 10^6spores or log-phase (vegetative) bacteria with or without Cry1C toxin or with toxin alone. The larvae were incubated at 37 °C, and mortality was scored 24 h after force-feeding. Results: No differences were observed between the wildtype HD73 and the CBPA mutant strain. No mortality was observed with bacteria or spores alone and high mortality (80% to 95% was observed upon addition of Cry1C, indicating clear synergism, as reported previously with two other Bt and *B. cereus* strains (Salamitou *et al*. 2000).
